# Idiopathic Pulmonary Fibrosis Complicated by Adenocarcinoma and Organizing Pneumonia

**DOI:** 10.7759/cureus.20916

**Published:** 2022-01-04

**Authors:** Yu Inutsuka, Toyoshi Yanagihara, Kotaro Matsumoto, Reiko Yoneda, Mikiko Hashisako, Naruhiko Ogo, Tatsuma Asoh, Takashige Maeyama

**Affiliations:** 1 Respiratory Medicine, Hamanomachi Hospital, Fukuoka, JPN; 2 Surgery, Hamanomachi Hospital, Fukuoka, JPN; 3 Pathology, Hamanomachi Hospital, Fukuoka, JPN; 4 Department of Pathology, Kyushu University, Fukuoka, JPN

**Keywords:** usual interstitial pneumonia, lung cancer, organizing pneumonia, adenocarcinoma, idiopathic pulmonary fibrosis

## Abstract

We describe a case of a 77-year-old male with idiopathic pulmonary fibrosis (IPF) complicated by lung adenocarcinoma and organizing pneumonia (OP). On initial examination, physical examination revealed fine crackles in both sides of his chest. There were no physical findings suggestive of collagen disease. Blood tests showed no elevation of C-reactive protein, and lactate dehydrogenase and Krebs von den Lungen-6 (KL-6) were within normal limits. A high-resolution CT (HRCT) of the chest showed multiple ground-glass opacities (GGOs) in both lungs, with consolidation and traction bronchiectasis in the left lower lobe. Although a bronchoscopy was performed, no diagnosis could be made. Bronchoalveolar lavage showed elevated lymphocytes, and treatment with prednisolone was started for the possibility of OP. Subsequent chest X-ray and chest CT showed worsening of the shadows over time, and shortness of breath on exertion progressed. Surgical lung biopsy revealed IPF complicated by adenocarcinoma and OP. Although the patient was treated with pemetrexed and carboplatin combination therapy, respiratory failure progressed, and palliative care was decided. There is no report of IPF complicated by adenocarcinoma and OP, and early surgical lung biopsy may be important for diagnosis.

## Introduction

Idiopathic pulmonary fibrosis (IPF) is a chronic, progressive interstitial lung disease (ILD) of unknown etiology, occurring mainly in the elderly [1]. Focused history taking, physical examination, and serological tests should be performed to identify known causes of interstitial lung diseases (ILDs) [2]. If the cause of ILD is still not identified and a high-resolution CT (HRCT) shows a characteristic imaging finding called the usual interstitial pneumonia (UIP) pattern, IPF can be diagnosed [2,3]. However, it is difficult to diagnose in some cases, and IPF is sometimes misdiagnosed as heart failure or chronic obstructive pulmonary disease (COPD) [4]. IPF can be complicated by lung cancer, and there have been reports of lung cancer and organizing pneumonia (OP) [5-11]. Diagnosis becomes very difficult when multiple diseases are involved. If an accurate diagnosis cannot be made, early treatment cannot be given, and the time for treatment is missed. In this article, we describe a case of IPF complicated by adenocarcinoma and OP.

## Case presentation

**Figure 1:**
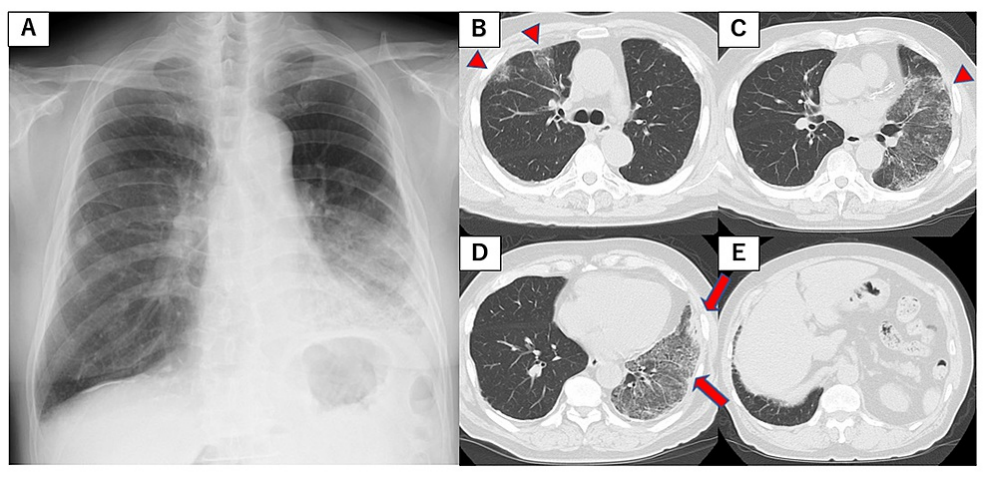
Radiological findings at initial examination. (A) Chest X-ray image. (B–E) Chest CT images showing multiple ground-glass opacities (GGOs) in both lungs (arrowheads), with consolidation and traction bronchiectasis in the left lower lobe (arrows).

A 77-year-old male was referred to Hamanomachi Hospital for coughing for several weeks. His medical history comprised left lower lobe pneumonia two years earlier, which resolved spontaneously. He was a past smoker with 90 pack-years. He had a temperature of 36.5°C, blood pressure of 165/85 mmHg, a heart rate of 84 beats per minute, and oxygen saturation of 97% under room air. His Eastern Cooperative Oncology Group (ECOG) performance status was 1. Physical examination revealed fine crackles in both sides of his chest. There were no physical findings suggestive of collagen diseases. Blood tests showed no elevation of C-reactive protein, and lactate dehydrogenase and Krebs von den Lungen-6 (KL-6) were within normal limits. Carcinoembryonic antigen (CEA) and CYFRA21-1 were within the reference values. The levels of rheumatoid factor, antinuclear antibody, and anti-aminoacyl tRNA synthetase were negative. A high-resolution CT (HRCT) of the chest showed multiple ground-glass opacities in both lungs, with consolidation and traction bronchiectasis in the left lower lobe (Figure 1).

There was no obvious lymph node enlargement. Although clarithromycin 200 mg and vonoprazan 20 mg were started for the possibility of chronic bronchitis and bronchiectasis due to aspiration or some other cause, no improvement was observed. A bronchoscopy was performed to make a diagnosis. A transbronchial lung biopsy (TBLB) was performed from the left B8. Bronchoalveolar lavage fluid (BALF) was obtained from the left B5, where GGO was observed. TBLB showed mild inflammatory cell infiltration without malignant findings or suspicious findings of organizing pneumonia (OP). BALF not only showed alveolar macrophage predominance but also elevated lymphocytes and neutrophils (45% macrophages, 27% neutrophils, 25% lymphocytes, and 3% eosinophils).

**Figure 2:**
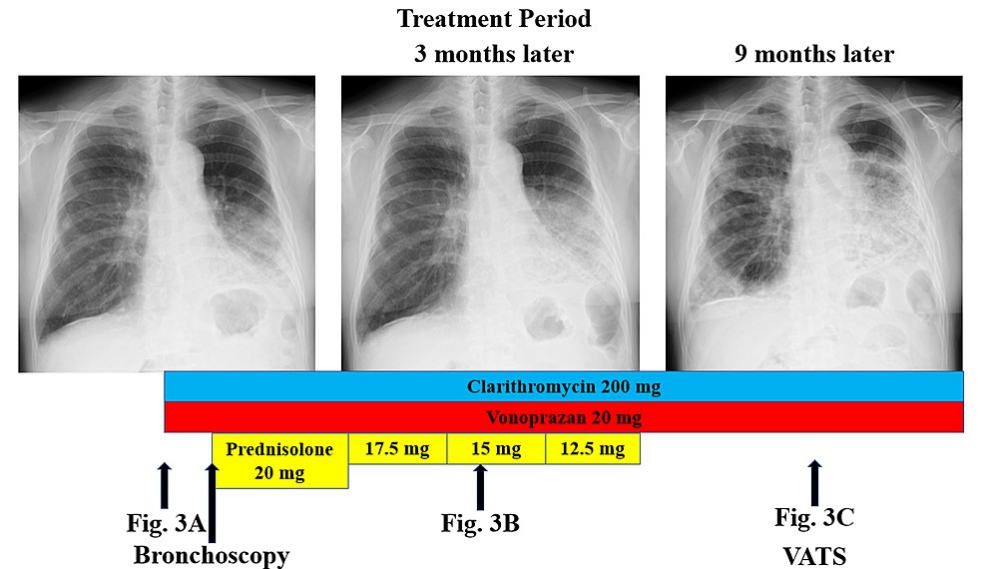
Clinical course of the patient. Although clarithromycin 200 mg and vonoprazan 20 mg were started for the possibility of chronic bronchitis and bronchiectasis due to aspiration or some other cause, no improvement was observed. BALF from left B5 showed elevated lymphocytes, and treatment with prednisolone 20 mg was started for the possibility of organizing pneumonia. The chest X-ray showed multiple GGOs that spread over time, and shortness of breath on exertion progressed.

**Figure 3:**
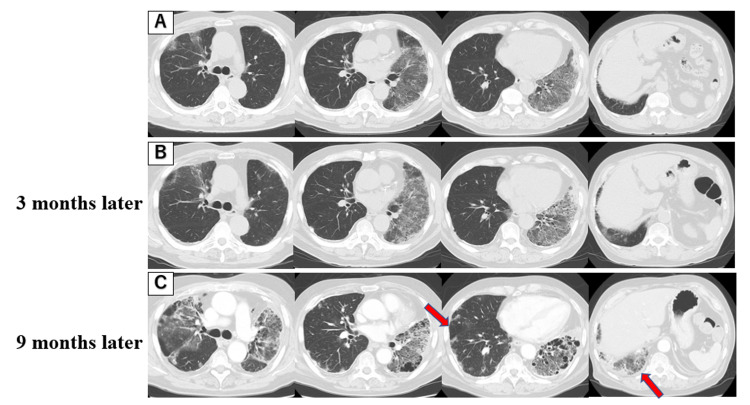
Chest CT images of the patient over time and location of partial lung resection. The resection site was described by the bronchopulmonary segments (S1-10). (A–C) The chest CT showed multiple GGOs that spread over time. Partial lung resection was performed from two locations (arrows): from the right middle lobe (S4), which was near normal grossly, and the right lower lobe (S10), which was highly fibrotic.

Based on the lymphocytosis in BALF, treatment with prednisolone 20 mg was initiated for the possibility of OP (Figure 2). Although the patient’s cough had mildly improved, subsequent chest X-ray and chest CT imaging showed multiple GGOs that spread over time, and shortness of breath on exertion progressed (Figure 2, Figure 3).

Prednisolone was tapered off and terminated after about three months due to lack of efficacy. Although there was a risk of acute exacerbation of interstitial lung disease, video-assisted thoracic surgery (VATS) was performed because the cause of the disease was unknown, and a treatment plan could not be determined. Partial lung resection was performed from two locations (Figure 3).

**Figure 4:**
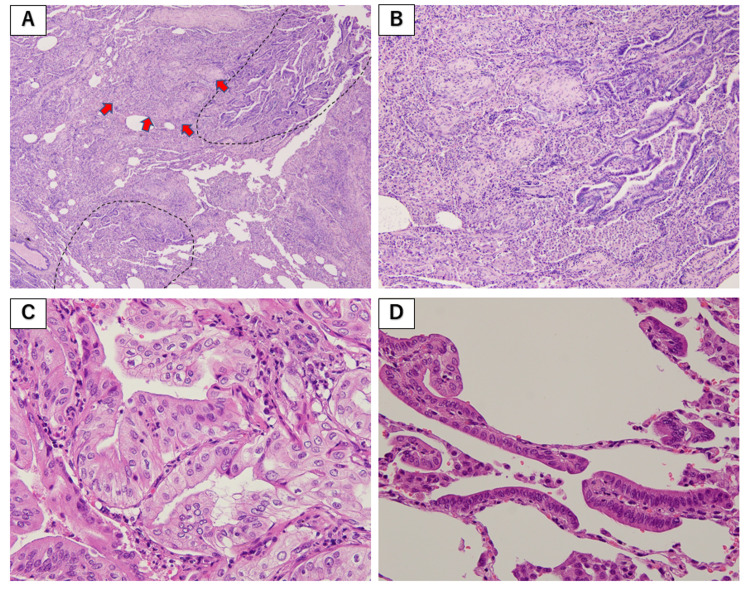
Pathological findings from the right middle lobe (S4). Hematoxylin and eosin staining: (A) ×40, (B) ×100, and (C,D) ×400. (A,B) Adenocarcinoma (dotted lines) and OP (arrows). (C) Acinar-predominant adenocarcinoma. (D) Lepidic-predominant adenocarcinoma.

It was performed from the right middle lobe (S4), which was near normal grossly, and the right lower lobe (S10), which was highly fibrotic. The pathological findings from S4 showed acinar-predominant adenocarcinoma, and a small amount of lepidic-predominant adenocarcinoma was also observed in the surrounding area (Figure 4). There was also OP and thickening of alveolar septa around the carcinoma.

**Figure 5:**
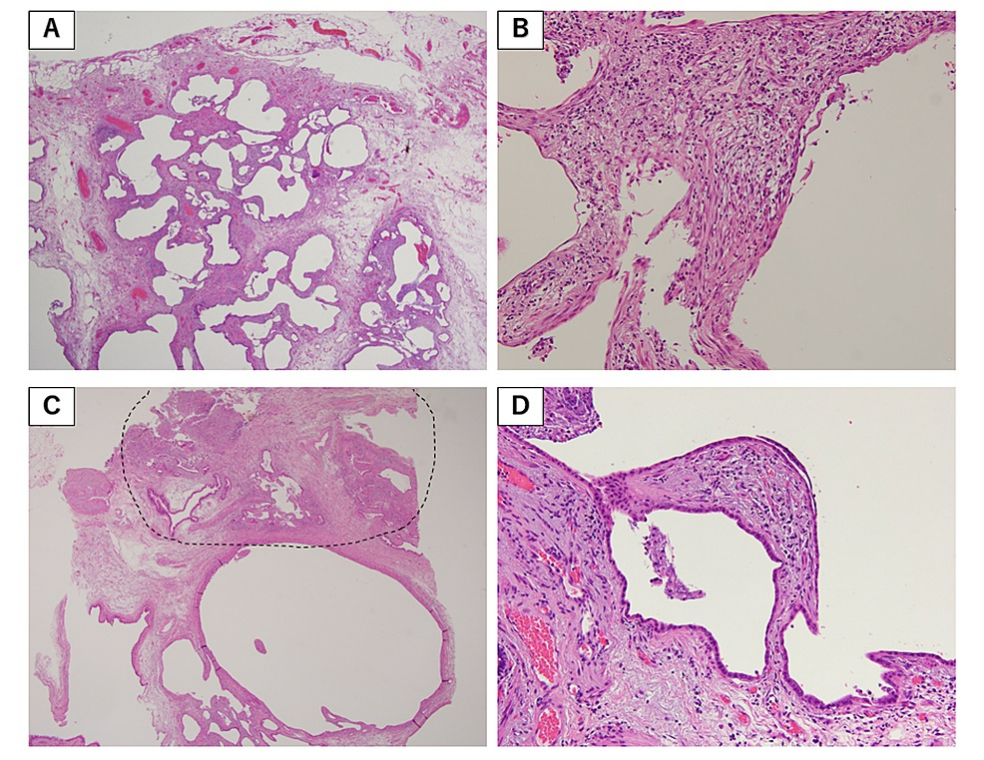
Pathological findings from the right lower lobe (S10). Hematoxylin and eosin staining: (A,C) ×20 and (B,D) ×400. (A) Probable UIP pattern. (B) Fibroblastic foci. (C,D) Atypical cells (dotted line).

The pathological findings from S10 showed dense fibrosis with architectural distortion, patchy involvement of lung parenchyma by fibrosis, and fibroblastic foci (Figure 5). Pathologically, probable UIP was observed according to ATS/ERS/JRS/ALAT clinical practice guidelines [3]. There were also atypical cells that were highly suspicious for adenocarcinoma. HRCT also showed subpleural and basal predominant reticular pattern and peripheral traction bronchiectasis, which was radiologically probable UIP. Based on HRCT findings and histopathology patterns, we diagnosed IPF [3].

To determine the stage of lung cancer, contrast-enhanced magnetic resonance imaging (MRI) scan of the head, bone scintigraphy, and contrast-enhanced CT scan of the neck, chest, abdomen, and pelvis were performed. Contrast-enhanced MRI showed small enhancing lesions in two locations, the right frontal lobe and posterior cervical spinal cord, which was determined to be a metastasis. Bone scintigraphy showed no evidence of bone metastasis. The contrast-enhanced CT scan showed no evidence of distant metastasis except for lung metastases. Based on the clinical course and pathological findings, the final diagnosis was adenocarcinoma of the lung (cT4N0M1c stage 4B, EGFR (-), ALK (-), ROS1 (-)), OP secondary to lung adenocarcinoma, and IPF. Because of the complication of IPF, we did not use immune checkpoint inhibitors and treated with pemetrexed and carboplatin combination therapy. Four days after the start of chemotherapy, worsening of respiratory failure and new bilateral ground-glass opacity on the chest X-ray were observed, and acute exacerbation of interstitial pneumonia was diagnosed. Although methylprednisolone pulse therapy was started, respiratory failure progressed. It was thought that it would be difficult to continue chemotherapy, and palliative care was decided.

## Discussion

We report a case of IPF complicated by lung adenocarcinoma and OP. Lung cancer often complicates with IPF, and the prevalence of lung cancer in patients with IPF has been reported to range from 4.4% to 48% [5,6]. It has been reported that 135 (14.5%) of 938 patients with IPF developed lung cancer during the follow-up period from 1998 to 2013 at a Korean institution [7]. The patients who developed lung cancer were more likely to be men with a history of smoking [1,7]. Several studies have shown the possible mechanisms of carcinogenesis in patients with IPF. Honda et al. investigated 691 Japanese patients with lung adenocarcinoma, of whom 54 had UIP [12]. The authors found that the EGFR mutation was markedly less prevalent in patients with UIP than in those without UIP. Pulmonary surfactant system genes such as TTF1, SFTPA1, SFTPA2, SFTPB, and SFTPC were identified as targets for mutations, and these mutations were specifically associated with shorter overall survival (OS) of patients with lung adenocarcinoma complicated with UIP, independent of pathologic stage [12]. Since gene mutations in the pulmonary surfactant system can cause not only pulmonary fibrosis but also cancer [13,14], it may be reasonable that these mutations have carcinogenic potential. Otsubo et al. analyzed 17 IPF-associated non-small cell lung cancer (NSCLC) and 15 paired fibrosing lung tissue specimens [15]. The authors discovered that gene mutations in the RAS-RAF pathway were found in both tumor samples and fibrosing lung tissue specimens [15].

It has also been reported that OP is sometimes associated with lung cancer [8-10]. Although the pathogenesis for OP is largely unknown, various mechanisms have been assumed, including obstruction, speciﬁc tumor factors, and local host response to the tumor mediated by the immune system [11]. In this case, the inflammation caused by the tumor combined with the fibrosis associated with IPF may have predisposed the patient to develop OP. The pneumonia that resolved spontaneously two years earlier may have been OP secondary to lung cancer. On the other hand, it has been reported that the histological type of non-mucin-producing adenocarcinoma with consolidation on the chest radiography is often of acinar or lepidic pattern, which is consistent with this case [11]. In this case, the pathology showed conspicuous OP around the adenocarcinoma, which was thought to be one of the reasons for extensive ground-glass opacities on the chest CT. Although OP is generally well responsive to glucocorticoid therapy, there are reports of poor responsiveness in OP secondary to lung cancer [16]. In this case, glucocorticoid therapy did not improve clinical symptoms and multiple GGOs on the chest X-ray.

Although there are no reports of IPF complicated by adenocarcinoma and OP, histopathology is essential for definitive diagnosis, and surgical lung biopsy is required. Since surgical lung biopsy is associated with the risk of acute exacerbation of IPF, it can be assumed that there were simply no cases that could be diagnosed as in this case. It is also possible that the pathology of organizing pneumonia around lung cancer may be underdiagnosed.

There are no guidelines for chemotherapy for lung cancer complicated by idiopathic interstitial pneumonias (IIPs). However, there are several reports indicating that chemotherapy may be effective for patients with NSCLC complicated by IIPs [17-19]. Minegishi et al. prospectively evaluated the safety and efficacy of weekly paclitaxel in combination with carboplatin as first-line therapy for 18 patients with NSCLC complicated by IIPs. Acute exacerbation of IIPs occurred in one patient (5.6%), and the overall response rate (ORR) was 61%. The median progression-free survival (PFS) was 5.3 months, and the median survival time (MST) was 10.6 months [19]. Nintedanib, a multi-targeted tyrosine kinase inhibitor, is also used to treat IPF. The results of several trials, including the TOMORROW trial and the INPULSIS trial, have shown that nintedanib reduces lung function decline and acute exacerbations in patients with IPF [20,21]. There is also a study investigating nintedanib in the treatment of non-small cell lung cancer (NSCLC). The LUME-Lung 1 study, a phase 3, double-blind, randomized controlled trial (RCT), evaluated the efficacy and safety of docetaxel plus nintedanib as second-line chemotherapy for NSCLC [22]. This study showed that the docetaxel plus nintedanib group significantly prolonged progression-free survival (PFS) compared with the docetaxel plus placebo group. In addition, the docetaxel plus nintedanib group had prolonged overall survival (OS) compared with the docetaxel plus placebo group in patients with adenocarcinoma [22]. Recently, the J-SONIC study, a randomized study of carboplatin plus nab-paclitaxel with or without nintedanib for NSCLC with IPF, has been conducted [23]. Such a treatment may be an option, especially for the treatment of lung cancer complicated by IPF. In this case, the patient was treated with pemetrexed and carboplatin combination therapy. There are few reports of carboplatin-related interstitial lung disease (ILD), and the risk of pemetrexed-related ILD is reported to be approximately 3.6% [24,25]. These numbers are not significantly higher than those of other anticancer agents [25]. Although the risk of acute exacerbation in NSCLC complicated by IIPs is estimated to be even higher, we thought that chemotherapy treatment for lung cancer might be beneficial for the patient compared to best supportive care. If it had been possible to diagnose the disease early, chemotherapy with nintedanib could have been considered. Although there is a risk of acute exacerbation with surgical lung biopsy, it may be necessary for diagnosis in atypical cases, even in the elderly.

## Conclusions

In conclusion, adenocarcinoma and OP are rare complications of IPF and are often overlooked. Complications of multiple lung diseases may lead to an atypical course, in which case surgical biopsy should be considered even in elderly patients. Although there are no guidelines for chemotherapy for lung cancer complicated by IIPs, several reports are indicating that chemotherapy may be beneficial. Chemotherapy with nintedanib is particularly expected for lung cancer complicated by IPF.
